# Efficacy of Fetal Wharton’s Jelly Mesenchymal Stem Cells-Derived Small Extracellular Vesicles in Metabolic Syndrome

**DOI:** 10.3390/biom15010044

**Published:** 2025-01-01

**Authors:** Illayaraja Krishnan, Magdalene Tan Mei Ling, Min Hwei Ng, Jia Xian Law, Mohd Rafizul Mohd Yusof, Thavachelvi Thangarajah, Zalina Mahmood, Nurul Izzati Uda Zahli, Shathiya Rajamanickam, Baskar Subramani, Yogeswaran Lokanathan

**Affiliations:** 1 Department of Tissue Engineering and Regenerative Medicine (DTERM), Faculty of Medicine, Universiti Kebangsaan Malaysia (UKM), Cheras, Kuala Lumpur 56000, Malaysia; p114013@siswa.ukm.edu.my (I.K.); magtan97@gmail.com (M.T.M.L.); angela@ppukm.ukm.edu.my (M.H.N.); lawjx@ppukm.ukm.edu.my (J.X.L.); 2 Department of Clinical Pharmacy and Pharmacy Practice, Faculty of Pharmacy, Universiti Malaya (UM), Kuala Lumpur 50603, Malaysia; 3 Department of Parasitology and Medical Entomology, Faculty of Medicine, Universiti Kebangsaan Malaysia (UKM), Cheras, Kuala Lumpur 56000, Malaysia; rafizulyusof@ukm.edu.my; 4 Department of Obstetrics and Gynaecology, Hospital Angkatan Tentera (HAT) Tuanku Mizan, Wangsa Maju, Kuala Lumpur 53300, Malaysia; chelvi777@yahoo.com; 5 Production and Blood Supply Management Division, National Blood Centre, Jalan Tun Razak, Kuala Lumpur 50400, Malaysia; dr.zalina@moh.gov.my; 6 Department of Veterinary Pathology and Microbiology, Faculty of Veterinary Medicine, Universiti Putra Malaysia (UPM), Serdang 43400, Malaysia; nurulizzati.udazahli@upm.edu.my; 7 Medixcell Sdn. Bhd., Level 5, Equatorial Plaza, Lot 5-5 & 5-6, Jalan Sultan Ismail, Kuala Lumpur 50250, Malaysia; shakthi_rj21@yahoo.com (S.R.); sudabas23@gmail.com (B.S.); 8 Advance Bioactive Materials-Cells UKM Research Group, Universiti Kebangsaan Malaysia, Bangi 43600, Malaysia

**Keywords:** umbilical cord, exosomes, metabolic diseases, syndrome X, biotherapeutics

## Abstract

Background/Objective: Metabolic syndrome (MetS) is characterized by abdominal obesity, increased blood pressure (BP), fasting blood glucose (FBG) and triglyceride levels, and reduced high-density lipoprotein (HDL) levels. This study aims to investigate the efficacy of the Wharton’s jelly mesenchymal stem cells (WJMSCs)-derived small extracellular vesicles’ (sEVs) preparations in managing MetS. Method: Twenty-four rats were fed with a high-fat and high-fructose diet to induce MetS for 16 weeks and randomized into three groups (*n* = 8/group): a MetS Control group treated with normal saline, MetS Low Dose (LD) group treated with a LD of sEVs preparations (3 × 10^9^ particle/rat), and MetS High Dose (HD) group treated with a HD of sEVs preparations (9 × 10^9^ particles/rat). The Control Non-Disease (ND) group was given a standard rat diet and autoclaved tap water with normal saline as treatment. Treatments were given via intravenous injection every three weeks for twelve weeks. Rats were assessed every six weeks for physical measurements, FBG, lipid profiles, CRP, leptin, adiponectin, and BP. Necropsy evaluation was performed on the lungs, liver, spleen, and kidney. Results: Significant reductions in FBG, triglycerides, BP, and increased HDL levels were observed in the treated groups compared to the control group. However, significant abdominal circumference (AC) improvement was not observed in the treated groups. Non-significant associations were found between fasting CRP, leptin, and adiponectin levels with MetS rats after treatment. In addition, sEVs preparations improved inflammation and hemorrhage in the lung and mineralisation in the renal of the treated group. Conclusions: Human fetal WJMSCs-derived sEVs preparations improve all the clusters of MetS in rats except AC and could be further explored as a treatment for MetS.

## 1. Introduction

Metabolic syndrome (MetS) is a cluster of metabolic dysfunctions conditions and is also known as Syndrome X or Reaven Syndrome [1]. This non-communicable syndrome is characterized by abdominal obesity, increased blood pressure (BP), increased fasting blood glucose (FBG), and dyslipidemia [2]. MetS prevalence is a worrying threat to worldwide public health concerns and the healthcare cost burden incurred [3]. This is due to the risks associated with MetS, such as cardiovascular-related ailments and type 2 diabetes mellitus [4], that results in many other complications, such as major organ failure in the long term if unattended immediately [5]. The leading cause of MetS is believed to be adipocyte dysfunctions and results in elevated oxidative stress, which causes low-grade inflammation, vascular dysfunction, and atherosclerosis [6]. Inflammation at the cellular level is a complicated and multifaceted pathophysiological condition involving many imbalance markers such as adipokines and interleukins [7].

Wharton’s jelly mesenchymal stem cells (WJMSCs) are a widely used source of mesenchymal stem cells (MSCs) due to their many advantages over other sources of MSCs, such as bone marrow (BM) and adipose tissue (AT). Briefly, WJMSCs are popular due to invasive collection, continuous supply during childbirth, maternity ‘waste’ unwanted product and readily available if consented, higher proliferation rate, higher differentiation capacity, self-renewal, less immunogenicity with strong immunomodulatory, and anti-inflammatory properties compared to other sources [8,9]. WJMSCs are obtained from the umbilical cord (UC) and, therefore, are fresh and young MSCs with no exposure to other harmful substances; they possess high proliferation capacity, relatively low immunogenicity, tumorigenicity, easily grow in vitro properties, and are collected via non-invasive protocols, which makes them the ideal choice for researchers in stem cell therapy [10]. In addition, adult MSCs such as BM and AT tend to reduce their expansion capacity due to cell senescence [11]. The most important properties of WJMSCs that make it a potential for the treatment of MetS are the anti-inflammatory properties besides the immunomodulation and regenerative function [12,13,14]. Different parts of the UC have different properties and qualities where the fetal WJMSCs have been reported to have anti-inflammatory properties compared to other parts of the UC such as the maternal and middle parts [15,16,17].

With the advancement in research, it was discovered that MSCs possessed their regenerative, immunomodulatory, and anti-inflammation properties through paracrine activities, a type of intercellular communication by releasing a ‘tiny’ soluble substance known later as extracellular vesicles (EVs) in the biological systems [18,19]. EVs provide a better profile than MSCs due to the limitations of the latter, such as the risk of thromboembolism, risk of malignancy, triggering an immune response in the host, and stability issues [20]. All these barriers from MSCs were not observed when using EVs, and they gained recognition and caused a shift in the research paradigm from cell therapy to cell-free therapy. EVs have been proven to play a significant role in delivering a variety of biological substances such as lipids, proteins, and nucleic acids in their cargo to the cells of the target via intercellular communication and cause the desired physiological activities [21].

Potential biotherapeutics agents such as EVs are subjected to efficacy assessment after the quality and safety have been thoroughly evaluated. This ensures that the EVs are effective for the intended disease and fulfill mandatory regulatory requirements when applying for clinical trials and marketing authorisation approval. Efficacy study is also a type of pharmacological evaluation that studies the action of EVs in biological systems using the intended disease model. Efficacy study is a part of the non-clinical stage of drug development and it mimics Phase II of the clinical trial, where the EVs will be tested on diseased human subjects once satisfactory quality, safety, and efficacy data are submitted for regulatory approval [22,23]. Efficacy data cover the pharmacodynamics aspect where EVs’ actions in biological systems are evaluated comprehensively. Simply put, an efficacy study shows the clinical pledge that EVs can deliver for a target disease [24].

The appropriate doses of EVs will determine the desired therapeutic outcomes, and the subtherapeutic doses may cause inaccurate interpretation of the efficacy study data. Before testing the chosen therapeutic dose(s) in the efficacy study, they must first be verified for toxicity in the safety study. In the case of EVs’ dosing, it has been reported that their biological response may be dose-dependent [25,26]. Testing the effectiveness of EVs using different doses provides more valuable insight into their multiple potential and a better understanding of how to predict their mechanism of action in the future.

An efficacy study using an animal model is preferred over an in vitro model due to the similarities between human and animal biological systems depending on the chosen animal model. In vitro efficacy study results are difficult to forecast and extrapolate to humans, especially in tissue pathological assessment. Creating complex disease models such as MetS in an in vitro system is also challenging. Any novel potential biotherapeutics such as EVs are best tested in vivo using appropriate and carefully designed animal studies [27]. Animal model selection takes account of the aim of the research, the hypothesis, and, of course, the research budget [28].

This study aims to test the efficacy of the characterized and standardized pooled fetal WJMSCs-derived sEVs preparations in managing MetS. Before this efficacy study, the pooled fetal WJMSCs-derived sEVs preparations were tested to ensure their quality and safety to be administered intravenously in rats. MetS induction was performed using the high-fat and high-fructose (HFHF) diet plan to establish the MetS animal model in rats. The treatment included two different doses of sEVs preparations: low dose and high dose. The high dose was tested in the safety study and found safe to be administered intravenously to healthy rats.

## 2. Study Design

**Figure 1:**
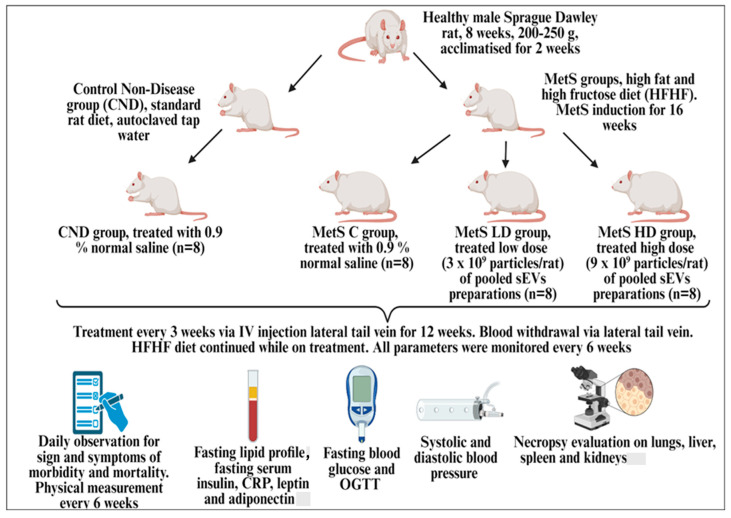
Study design.

The overall efficacy study design is illustrated in a brief graphical methodology in Figure 1 with 16 weeks of rat MetS model induction followed by 12 weeks of treatment and monitoring plan.

## 3. Materials and Method

### 3.1. Fetal Umbilical Cord Protocol and Isolation of Small Extracellular Vesicles

The fetal part of the human UC was used to isolate fetal WJMSCs. These fetal UCs were collected from healthy-term pregnant mothers with informed consent forms from the Department of Obstetrics & Gynaecology at Hospital Angkatan Tentera (HAT) Tuanku Mizan, Wangsa Maju, Ministry of Defense, Kuala Lumpur, via either spontaneous vaginal delivery or cesarean section. Inclusion criteria for UC donation involve the donor being over 18 years old, having a gestational age of 37–40 weeks, having no medical complications, and not being under any medications. Pregnant mothers who tested positive for any transmissible infectious diseases like human immunodeficiency virus (HIV), hepatitis virus types B and C, and syphilis were excluded from this study [29,30]. A general protocol was provided below while the preparation of the manuscript was in progress.

#### 3.1.1. Isolation and Culture of Fetal UCMSCs

After collection, the four independent UCs were transferred in sterile conditions to the laboratory within 24 h for processing. The sample processing and isolation of fetal WJMSCs were performed as previously reported [31]. The local arteries and veins were carefully excised, and the remaining white parenchyma was shredded into thin strips (0.5–1.0 mm^2^). The tissue was digested in 0.6% Collagenase Type 1 (Worthington, OH, USA) and incubated in a 37 °C shaker incubator for one hour with continuous monitoring every 20 min to ensure the samples were not over-digested. The complete culture medium of alpha Minimum Essential Medium (α-MEM) was prepared by mixing 1% (*v*/*v*) Glutamax (Gibco, Remote, Germany), 1% (*v*/*v*) HEPES (Gibco, Germany), 1% (*v*/*v*) antibiotic-antimycotic (Gibco, Germany), and 10% (*v*/*v*) in-house human platelet lysate (hPL) with the basal α-MEM culture medium. The hPL was prepared in-house as previously reported [32]. This complete culture medium was added in equal volume with the digested fetal WJMSCs suspension to neutralise the collagenase activity. Following centrifugation at 5000 rpm for 5 min, the supernatant was discarded, and the pellet was resuspended in a complete medium. The cells were seeded at an initial 3000 cells/cm^2^ density and maintained in a 37 °C incubator with 5% CO_2_. The culture medium change was performed after the first 24 h and thereafter every three days. At 90% confluency, the cells were harvested using TrypLe Express Enzyme (Gibco, Germany) and subcultured. By passage 1, the cells were enumerated and cryopreserved at 1 million cells per cryovial.

#### 3.1.2. Characterization of Fetal WJMSCs

Fetal WJMSCs characterisation was performed at passage 3 according to the ISCT guidelines [33]. Morphology of the fetal UCMSCs was observed under an inverted light microscope (Olympus CK30 Light Microscope, Olympus, Hamburg, Germany). The MSCs positive markers (CD73, CD90, and CD105) and negative markers (CD11b, CD19, CD34, CD45, and HLA-DR) were identified through flow cytometry (BD FACSVerse™, Piscataway, NJ, USA) using the Human MSC Analysis Kit (BD Biosciences, San Jose, CA, USA) following the manufacturer’s protocols. Trilineage differentiation of UCMSCs for osteogenesis, adipogenesis, and chondrogenesis was confirmed using the StemPro™ (Osteogenesis/Chondrogenesis/Adipogenesis) Differentiation Kit (Gibco, Germany) following the manufacturer’s protocol. The differentiation medium was changed every two to three days. After continuous induction for 14–21 days, the cells were fixed with 4% (*w*/*v*) paraformaldehyde and stained with oil red O (Sigma-Aldrich, Rockville, MD, USA), Alizarin red (Sigma-Aldrich, USA) and Safranin O (Sigma-Aldrich, USA) to detect lipid droplets in adipogenic differentiated cells, calcium deposition in osteogenic differentiated cells, and glycosaminoglycans in chondrogenic differentiated cells, respectively. Fetal WJMSC characterization was performed on four independent samples separately.

#### 3.1.3. Isolation of sEVs

At passage 3, the four independent fetal WJMSCs were initially seeded at a seeding density of 3000/cm^2^ in a culture flask. The culture medium change was performed every three days. When the fetal WJMSCs reached 70–80% confluency, the culture medium was discarded, and the cells were gently washed twice with Phosphate-Buffer Saline (Sigma Aldrich, USA) before being replaced with a phenol red-free DMEM-LG (Sigma Aldrich, USA) basal medium. After 24 h, the conditioned medium (CM) was collected and transferred into 50 mL conical tubes. The CM was centrifuged at 2000× *g* for 15 min at 4 °C to remove cells and cell debris. Then, the supernatant of the CM was collected immediately and frozen at −80 °C until further use. The tangential flow filtration (TFF) method was used to isolate and concentrate the sEVs preparations from the CM. Thawed CM was used to isolate sEVs using the Minimate™ TFF system and Minimate™ TFF capsule with Omega™ polyethersulfone (PES) ultrafiltration 100 kDa membrane (Pall Corporation, Port Washington, NY, USA). The enriched sEVs preparations were sterile-filtered through a 0.22 μm filter and stored at −80 °C until further use.

#### 3.1.4. Characterisation of sEVs

The sEVs preparations were characterised based on minimal information for studies of extracellular vesicles 2018 (MISEV2018) guidelines [34] published by the International Society for Extracellular Vesicles (ISEV). Protein concentration was determined using Pierce™ BCA Protein Assay Kits (Thermo Fisher Scientific, Vacaville, CA, USA) following the manufacturer’s protocol. Particle size and distribution analysis were performed using nanoparticle tracking analysis (NTA) with NanoSight NS300 (Malvern Panalytical, Malvern, Worcestershire, UK). The sEVs protein markers were analysed for positive markers (CD63 and TSG101), negative markers (Grp94), and purity control (albumin) using Western blot analysis. The presence of bilipid layer sEVs and morphology were confirmed with transmission electron microscopy (TEM) analysis (Leo Libra 120, Carl Zeiss, Jena, Germany).

#### 3.1.5. Pooled sEVs Preparations

The four independent characterized sEVs preparations from the four independent fetal WJMSCs were pooled together and suspended in 0.9% normal saline when wanted to administer intravenously into the rat’s tail lateral vein (prepared freshly).

### 3.2. Animal Management

Thirty-three male-specific pathogen-free Sprague Dawley (SD) rats weighing approximately 200–250 g at eight weeks old were obtained from the Animal Experimental Unit (AEU), Universiti Malaysia, Kuala Lumpur. The rats were housed individually in ventilated polycarbonate cages (Allentown Inc., Allentown, NJ, USA) at room temperature of 22 °C with 12 h light and 12 h dark cycles. All the rats were acclimatised for two weeks before starting the experiment. The rats were fed with standard normal diet (SND) (Altromin 1314, Lage, Germany) and autoclaved tap water ad libitum for the randomly separated eight rats for the control group and the remaining twenty-five rats’ standard diet and autoclaved tap water was slowly acclimatised by mixing with HF diet (Altromin C 1090–70; Lage, Germany) and 30% (*w*/*v*) crystalline fructose (ADM Besin, Sarıçam/Adana, Turkey) solution for the MetS group within the two weeks acclimatisation period.

### 3.3. Animal Induction of MetS (−16 to 0 Weeks)

After the two-week acclimatization period, the HFHF group (*n* = 25) was fed a complete HF pellet and 30% (*w*/*v*) of fructose solution. The remaining rats in the control group (*n* = 8) were assigned to the Control ND group, which received SND and autoclaved tap water. Both diets were given ad libitum for 16 weeks. All the rats were monitored for 16 weeks for the presence of MetS and if they met three out of five MetS criteria: increased abdominal circumference (AC), hypertension, hyperglycemia, hypertriglyceridemia, and reduced high-density lipoprotein (HDL). Details of the monitoring as explained in the parameters for animal study are noted under Section 3.5.

### 3.4. Animal Treatment of MetS (0–12 Weeks)

Once the MetS animal model was successfully established after 16 weeks, all the HFHF group rats were randomly and equally assigned (*n* = 8/group) to three different treatment groups. The three groups were MetS control (MetS Control), MetS Low Dose (MetS LD), and MetS High Dose (MetS HD). The MetS Control group was given 0.9% normal saline (as a placebo), and both the MetS LD and MetS HD were given 3 × 10^9^ particles/rat and 9 × 10^9^ particles/rat of pooled fetal WJMSCs-derived sEVs preparations in 0.9% normal saline, respectively. A veterinarian gave the treatment via the rats’ lateral tail vein intravenously. The duration of the efficacy study was 12 weeks, and the treatment was given every three weeks up to nine weeks: four intravenous (IV) treatments were given. No treatment was given to all the rats from week 9 to week 12. Isoflurane (Piramal Critical Care, Mumbai, India) and oxygen were used with the anesthetic machine (Drager anesthesia machine, model NAD 2C, Belvidere, IL, USA) as an inhaled anesthetic agent for this procedure. All the rats were monitored as per the schedule provided in Table 1. Blood was collected via the rat’s lateral tail vein under isoflurane sedation. Daily observations for signs and symptoms of mortality and morbidity were performed and recorded throughout the 12 weeks of the study. In week 12, all the rats were euthanized by chemical method, an overdose via intraperitoneal (IP) administration of 2 mL (200 mg/mL) pentobarbital sodium (Vetoquinol, Towcester, UK). Necropsy and histological assessment were performed accordingly.

### 3.5. Parameters for Animal Study

#### 3.5.1. Morbidity and Mortality Observations

The rats were observed daily for signs and symptoms of mortality or morbidity from the start of the MetS induction period. These observation criteria were adopted from ‘Endpoint Guidelines for Animal Use Protocols’ (2018) drafted by the University of Maryland School of Medicine [35]. These criteria were approved for use by the Institutional Animal Care and Use Committee (IACUC) and also used in previous research [36] as described below.

Severe weight loss from anorexia and/or dehydration.
Dyspnoea (laboured breathing, hyperventilation, and abdominal distension).
Prolonged hypothermia or hyperthermia (palpable temperature).
Stress and/or poor grooming (rough stained coat and porphyrin built around nose and eyes).
Lethargy, hunched posture and inability to rise or ambulate.
Poor reflex or irresponsiveness to external stimuli.
Tumour growth.

#### 3.5.2. Physical Measurements

The rats were restrained with minimum force using plastic decapicones without using any sedation agents. Body weight (BW) was measured using a weighing scale, body length (BL) from nose to anus, and the abdominal circumference (AC) was measured using a measurement tape. The body mass index (BMI) of the rats was calculated by the ratio of BW (g) to BL squared (cm^2^). Food consumption was measured by the weight (g) of each rat’s diet consumed per week. Fluid intake was measured by the volume (mL) of fluid consumed weekly. Physical measurements were performed in weeks −16, 0, 6, and 12.

#### 3.5.3. Blood Serum Analysis

All the rats were fasted overnight with ad libitum access to autoclaved tap water only and bled at the intervals of week −16, 0, 6, and 12 via the rat lateral tail vein under the sedation of isoflurane as an anesthetic agent. Whole blood samples were collected using BD Vacutainer^®^ Blood Collection Tubes (BD Biosciences, USA) and allowed to stand and clot at room temperature for at least 30 min. The serum was collected via centrifugation at 5000× *g* for 20 min at 4 °C, aliquoted in Eppendorf tubes, and stored in the freezer at −80 °C until further use. Serum biochemistry test was conducted in the Haematology Laboratory located at the Veterinary Laboratory Service Unit (VLSU), Universiti Putra Malaysia (UPM), Malaysia, for fasting lipid profile test (CHO, triglyceride, HDL, and LDL). The remaining serum was used in enzyme-linked immunosorbent assay (ELISA) testing.

#### 3.5.4. Fasting Blood Glucose and Oral Glucose Tolerance Test

All the rats were fasted overnight with ad libitum access to autoclaved tap water. FBG levels were measured using an ACCU-Check Performa glucometer (Roche Diagnostic, Indianapolis, IN, USA). After baseline measurement, the rats were given 50% (*w*/*v*) dextrose solution and 2 g/kg rat BW via oral gavage [37]. Stainless steel straight oral gavage, 16GA × 76 mm (Instech Laboratories™ Feeding tube, Fisher Scientific, USA), was used. The rats were bled at 0 (baseline), 30, 60, 90, and 120 min at the rat lateral tail vein. The blood glucose levels were measured and recorded. This procedure was performed at the week −16, 0, 6, and 12 intervals.

#### 3.5.5. Blood Pressure

Systolic BP (SBP) and diastolic BP (DBP) were measured using a non-invasive tail-cuff system (CODA, Kent Scientific Corporation, Torrington, CT, USA) in weeks −16, 0, 6 and 12. The rats were restrained using plastic decapicones without anesthesia and briefly warmed on a far infrared warming platform (Kent Scientific Corporation, USA) before BP measurements were performed. TCODA software (CODA Standard NIBP System Channel, Version 3.01) was programmed to measure five acclimatization cycles and ten experimental cycles, each spaced between 30 s to allow blood flow recovery. All data were exported into Microsoft Excel 2019 MSO 64-bit (Microsoft Corp., Redmond, WA, USA) for further analysis.

#### 3.5.6. Necropsy and Relative Organ Weight

At the end of the study, in week 12, all the rats were euthanized by a chemical method using 2 mL of pentobarbital sodium overdose given via IP. When the animals were determined to be no longer conscious via the toe-pinch response method, they were dissected at the abdomen to reveal their subcutaneous adipose layer. Then, the rats were further dissected to reveal the thoracic and abdominal cavity. Major organs were collected, such as the lungs, liver, spleen, and kidney. The veterinarian conducted a gross pathological evaluation of each organ, and the results were recorded accordingly. Organ mass of the lungs, liver, spleen, and kidney were measured immediately using a weighing scale. The organs’ relative weight (%) was calculated as the percentage by dividing organ weight with BW and multiplying by 100. All the harvested organs were preserved in a 10% neutral buffered formalin (Chemiz, Shah Alam, Malaysia) immediately and stored at room temperature for paraffin sectioning.

#### 3.5.7. Histopathological Analysis

Paraffin blocks embedded with organs were cut with a microtome at 5 µm, deparaffined with xylene, and stained with standard haematoxylin (Epredia^TM^, Portsmouth, NH, USA) and eosin (Epredia^TM^, USA) staining. A blinded veterinary pathologist observed the stained slides under an inverted light microscope for histopathological assessment using the histopathological scoring system. The overall specific differences and findings for each group (collectively) were tabulated for easy understanding. The reference for the histopathological scoring system can be found at https://ntp.niehs.nih.gov/atlas/nnl (accessed on 20 June 2024), and similar approaches were used in the previous study [36].

#### 3.5.8. ELISA (Insulin and CRP)

Fasting rat serum insulin and CRP were measured using fasting serum and ELISA kits. Insulin ELISA kit (Catalogue No. E-EL-R3034, Elabscience, Wuhan, China) and CRP ELISA kit (Catalogue No. E-EL-R0506, Elabscience, China) were used, and the test was performed according to the manufacturer’s protocol in weeks −16, 0, 6 and 12. The absorbance was measured at 450 nm on a spectrophotometric multi-well plate reader (Biotek^®^, Santa Clara, CA, USA) with Gen 5 software, version 2.09.

#### 3.5.9. Mechanistic Study (Leptin and Adiponectin)

Fasting rat serum leptin and adiponectin were measured using fasting serum and ELISA kits. Leptin ELISA kit (Catalogue No. M0B00B, R&D Systems Inc., Santa Clara, CA, USA) and Adiponectin ELISA kit (Catalogue No. E-EL-R3012, Elabscience, China) were used, and the test was performed according to the manufacturer’s protocol in week 12 only. The absorbance was measured at 450 nm on a spectrophotometric multi-well plate reader (Biotek^®^, Santa Clara, CA, USA) with Gen 5 software, version 2.09.

#### 3.5.10. IR HOMA Score

Insulin resistance Homeostasis Model Assessment (IR HOMA) score [38] was calculated using the FBG and fasting insulin level as provided by the formula below for weeks −16, 0, 6, and 12.

$$IR–HOMA\;\mathrm{Score}=\frac{Insulin\;(U/L)\times \;\mathrm{Blood}\;\mathrm{Glucose}\;(\mathrm{mmol}/L)}{22.5}$$

### 3.6. Statistical Analysis

The sample size for the rats per group was determined by Mead’s resource equation [39,40]. All statistical analysis was performed using GraphPad software (GraphPad Prism version 9, La Jolla, CA, USA). The quantitative data or graphical results were presented as mean ± standard error mean (SEM). Comparisons between the four treatment groups for physical measurements, BP, FBG, fasting serum insulin, and fasting lipid profile were conducted through mixed-design ANOVA with time-fixed inter-group analysis and were calculated via Tukey’s multiple comparisons test (*p* < 0.05). Endpoint comparisons, such as the relative weight (%) of the organs and fasting serum leptin and adiponectin, were performed using one-way ANOVA followed by Tukey’s multiple comparisons test (*p* < 0.05). Statistical significance was set at *p* ≤ 0.05. Time point (week) analysis within the same group was only performed on the five MetS parameters: BP, FBG, fasting serum triglycerides, fasting serum HDL, and AC. All group comparisons within the same time point (week) were performed on all parameters. Additionally, time point (minutes) analysis within the same group was only performed on OGTT parameters in weeks 6 and 12.

## 4. Results

### 4.1. MetS Induction of Rat

At the end of 16 weeks of the MetS induction period, we successfully developed MetS in 24 rats fed the HFHF diet. To be diagnosed as MetS, at least three out of five MetS criteria needed to be fulfilled [41]. Table 2 summarizes the MetS parameters and the range derived from the Control ND group (*n* = 8). Readings above the cut-off value (BP, FBG, AC, and triglyceride) and below the cut-off value (HDL) were determined as Mets. Table 3 summarizes the MetS score for all the HFHF rats at the end of the MetS induction period. Only one rat failed to develop MetS during this induction period, and this rat was excluded from the treatment experiment. Most of the HFHF rats had a MetS score of 4/5 (*n* = 16), and only five rats managed to achieve the full MetS score. All the rats that developed MetS were divided almost equally and randomly based on the MetS score into three MetS treatment groups: MetS Control, MetS LD, and MetS HD, with eight rats in each group.

### 4.2. Mortality, Morbidity, and Physical Measurement

Daily observation records for signs and symptoms of morbidity and mortality throughout the 12-week treatment period were tabulated in Supplementary Table S1. The Control ND group did not show any of these signs and symptoms. Most of the HFHF diet rats showed dyspnea and stress due to poor grooming. No tumour growth and prolonged increase/decrease in body temperature were observed for all the rats.

**Figure 2:**
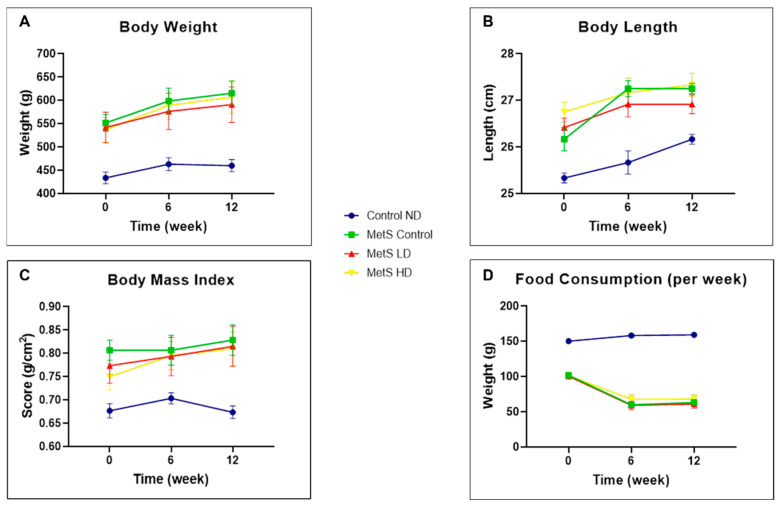
Physical measurements of (**A**) body weight, (**B**) body length, (**C**) body mass index, and (**D**) food consumption at weeks 0, 6, and 12. Data were presented as mean ± SEM (*n* = 8 rats per group). A difference at *p* ≤ 0.05 was considered statistically significant. Note: SEM: standard error mean.

**Figure 3:**
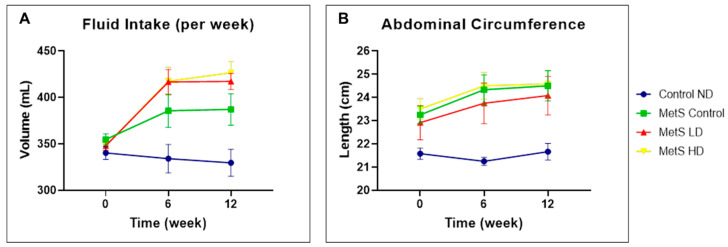
Physical measurements of (**A**) fluid intake and (**B**) abdominal circumference at weeks 0, 6, and 12. Data were presented as mean ± SEM (*n* = 8 rats per group). A difference at *p* ≤ 0.05 was considered statistically significant. Weekly comparison within the same group was only performed for the MetS parameter (abdominal circumference). Note: SEM: standard error mean.

All the results for week −16 were provided as Supplementary Figure S1 Week −16. Physical measurement results are displayed in Figure 2 and Figure 3. Generally, the MetS groups have higher BW, BL, and BMI than the Control ND group due to the consumption of the HFHF diet. Among these parameters, only AC is the MetS parameter. Notably, a statistically significant reduction in AC was not observed in the sEVs-treated groups. Therefore, pooled fetal WJMSCs-derived sEVs preparations do not positively impact AC reduction in MetS rats.

### 4.3. Fasting Lipid Profile

**Figure 4:**
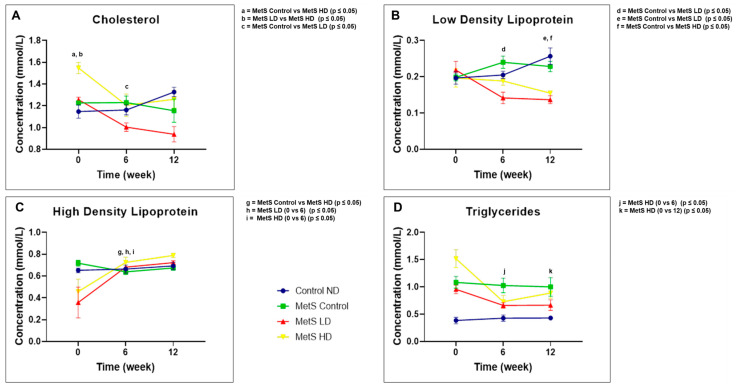
Fasting lipid profile showing (**A**) cholesterol, (**B**) LDL, (**C**) HDL, and (**D**) triglycerides at weeks 0, 6, and 12. Data were presented as mean ± SEM (*n* = 8 rats per group). A difference at *p* ≤ 0.05 was considered statistically significant. Weekly comparison within the same group only performed for the MetS parameter (HDL and triglycerides). Note: SEM: standard error mean; LDL: low-density lipoprotein; HDL: high-density lipoprotein.

The fasting lipid profile results are as shown in Figure 4. CHO level showed a statistically significant reduction (*p* ≤ 0.05) at week 6 between the MetS Control group and MetS LD group, which means that the low dose of the pooled fetal WJMSCs-derived sEVs preparations reduced the CHO level in MetS rats.

For the LDL level, a statistically significant reduction (*p* ≤ 0.05) was observed in week 6 between the MetS Control group and MetS LD group and in week 12 between the MetS Control group and both the sEVs-treated groups. Both low and high doses of the pooled fetal WJMSCs-derived sEVs preparations reduced the LDL levels after treatment in MetS rats.

HDL and triglycerides are both MetS parameters. HDL showed a statistically significant increased level (*p* ≤ 0.05) in week 12 between the MetS Control group and MetS HD group. The same group comparison with different time points showed no increased level statistically for the sEVs-treated groups. Therefore, high-dose sEVs preparations increased the HDL level after treatment in MetS rats. Triglycerides level showed no statistically significant reduction level for the sEVs-treated group after the treatment. However, a comparison of the different time points within the same group showed a reduction in triglyceride level with a statistically significant value (*p* ≤ 0.05) for the Mets HD group in week 0 versus 6 and 0 versus 12. This showed that the high dose of the sEVs preparations reduced the triglycerides after treatment in MetS rats. Pooled fetal WJMSCs-derived sEVs preparations have positive outcomes for HDL and triglycerides parameters in MetS rats.

### 4.4. FBG and OGTT

**Figure 5:**
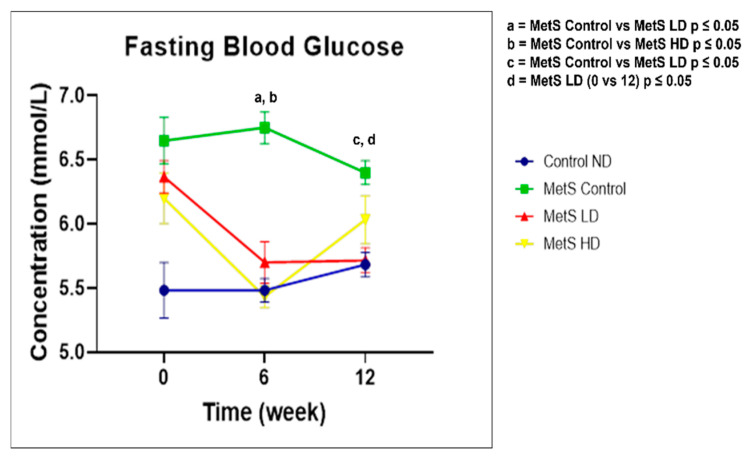
Fasting blood glucose. Data were presented as mean ± SEM (*n* = 8 rats per group) at weeks 0, 6, and 12. A difference at *p* ≤ 0.05 was considered statistically significant. Weekly comparison within the same group performed for fasting blood glucose since it is a MetS parameter. Note: SEM: standard error mean.

The FBG results are provided in Figure 5, and this is a MetS parameter. Comparison of the groups within the same time point showed a statistically significant reduction (*p* ≤ 0.05) in FBG at week 6 between the MetS Control group and both the treated groups. At week 12, a statistically significant reduction (*p* ≤ 0.05) was observed for the MetS Control group and MetS LD group. Comparison over the time period showed a statistically significant reduction (*p* ≤ 0.05) in FBG for the MetS LD group between weeks 0 and 12. These results showed that the low dose of the pooled fetal WJMSCs-derived sEVs preparations reduced the FBG in MetS rats.

**Figure 6:**
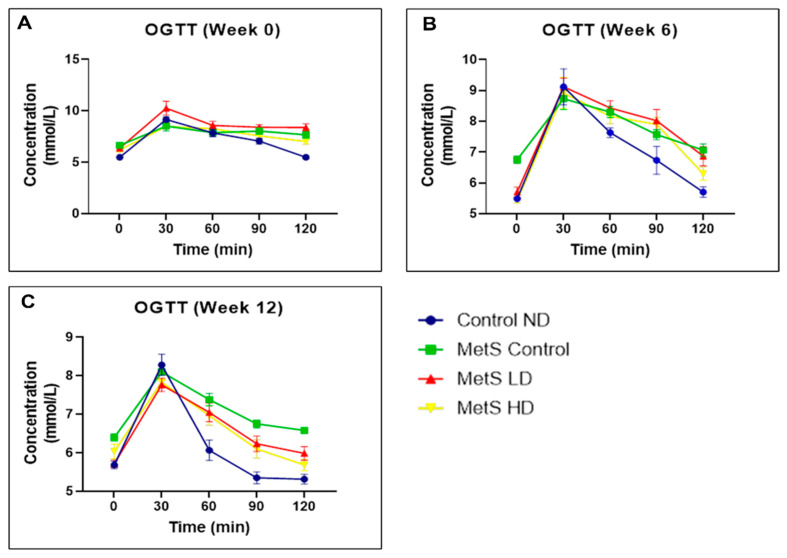
Oral glucose tolerance test at weeks (**A**) 0, (**B**) 6, and (**C**) 12. Data were presented as mean ± SEM (*n* = 8 rats per group). A difference at *p* ≤ 0.05 was considered statistically significant. Comparison within the same group at 0 versus 120 min was performed only for the OGTT parameter at weeks 6 and 12. Note: SEM: standard error mean.

The OGTT results are illustrated in Figure 6. No significant reduction in blood glucose level after 2 h was observed in the treated group at weeks 6 and 12. Based on these results, the pooled fetal WJMSCs-derived sEVs have no improvement in OGTT in the MetS rat.

### 4.5. Blood Pressure

**Figure 7:**
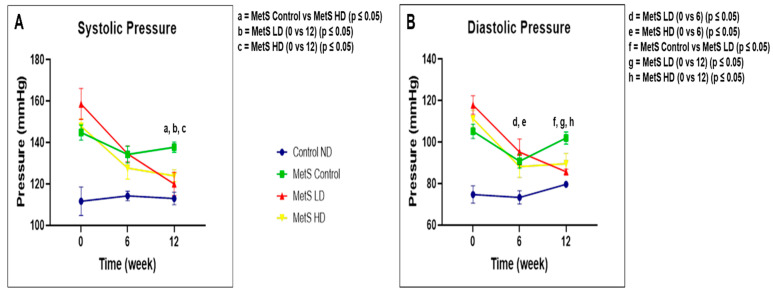
The (**A**) systolic and (**B**) diastolic blood pressure measurements at weeks 0, 6, and 12. Data were presented as mean ± SEM (*n* = 8 rats per group). A difference at *p* ≤ 0.05 was considered statistically significant. Weekly comparison within the same group performed for blood pressure since it is a MetS parameter. Note: SEM: standard error mean.

Both SBP and DBP results as illustrated in Figure 7. BP is a MetS parameter. For the SBP, a comparison of different groups at the same point showed a statistically significant reduction (*p* ≤ 0.05) at week 12 between the MetS Control group and the MetS HD group. Comparison of the same group with different time points showed that both the sEVs treated groups showed statistically significant reductions (*p* ≤ 0.05) in SBP at week 0 versus 12. DBP showed a statistically significant reduction (*p* ≤ 0.05) at week 12 between the MetS Control group and the MetS LD group. Comparing the group analysis over time showed a statistically significant reduction for both the treated groups at week 0 versus 6 (*p* ≤ 0.05) and 0 versus 12 (*p* ≤ 0.05). Therefore, pooled fetal WJMSCs-derived sEVs preparations reduced BP in MetS rats with low dose and high dose acting differently on both SBP and DBP at different time points.

### 4.6. Fasting Serum Insulin and IR HOMA Score

**Figure 8:**
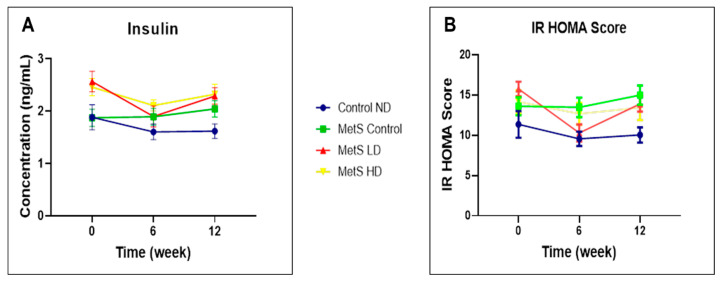
Fasting serum (**A**) insulin and (**B**) IR HOMA score at weeks 0, 6, and 12. Data were presented as mean ± SEM (*n* = 8 rats per group). A difference at *p* ≤ 0.05 was considered statistically significant. Note: SEM: standard error mean; IR HOMA: Insulin Resistance Homeostatic Model Assessment.

As shown in Figure 8, no statistically significant differences in either the fasting serum insulin levels or IR HOMA score were noted with the treatment groups. Based on these results, the pooled fetal WJMSCs-derived sEVs preparations have no impact on fasting insulin and IR HOMA score in MetS rats. MetS groups demonstrated normal insulin levels and IR HOMA scores comparable to the normal control.

### 4.7. Fasting Serum CRP, Leptin, and Adiponectin

**Figure 9:**
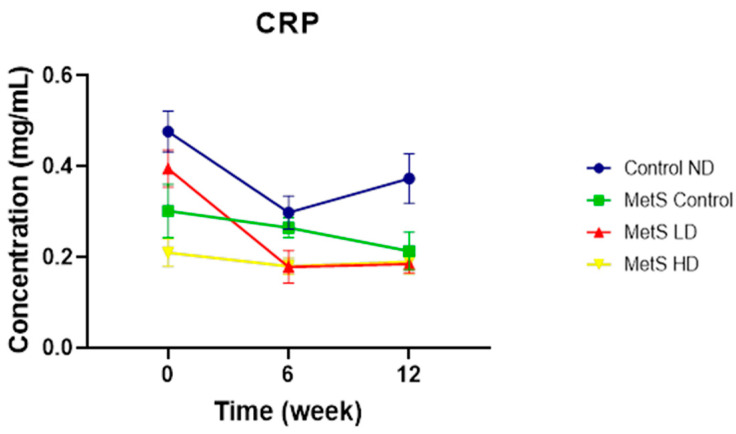
Fasting serum C-reactive protein level at weeks 0, 6, and 12. Data were presented as mean ± SEM (*n* = 8 rats per group). A difference at *p* ≤ 0.05 was considered statistically significant. Note: SEM: standard error mean.

No statistically significant results in a reduction in CRP were observed in the treated groups to conclude the positive effects of pooled fetal WJMSCs-derived sEVs preparations on fasting serum CRP levels in MetS rats, as shown in Figure 9.

**Figure 10:**
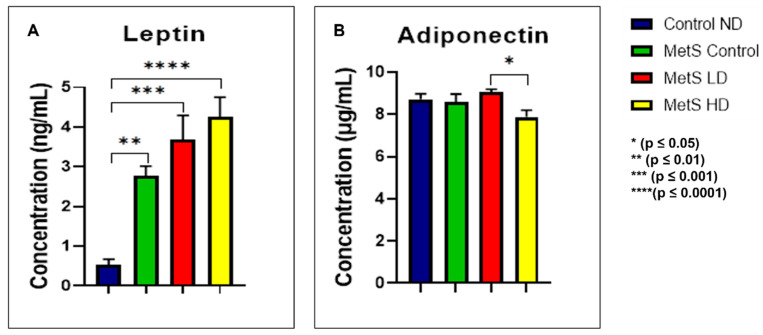
Fasting serum (**A**) leptin and (**B**) adiponectin at week 12. Data were presented as mean ± SEM (*n* = 8 rats per group). A difference at *p* ≤ 0.05 was considered statistically significant. Note: SEM: standard error mean.

Fasting serum leptin and adiponectin results were only measured at week 12, and these results are shown in Figure 10. For leptin, statistically significant (*p* ≤ 0.05) results were observed between the Control ND group and all the MetS groups. No significant relationship between the MetS Control and the sEVs-treated group was observed. Meanwhile, for adiponectin, a statistically significant (*p* ≤ 0.05) level was observed for both the sEVs-treated groups and non-statistically significant with the MetS Control group. Based on these limited data, preliminarily, it can be concluded that the pooled fetal WJMSCs-derived sEVs preparations have no impact on either the leptin or adiponectin in MetS rats.

### 4.8. Necropsy, Relative Organ Weight, and Histopathological Assessment

Table 4 shows the gross necropsy evaluation of the harvested organ at week 12. Mild congestion was observed in the lungs for the Control ND group and MetS LD group, with more incidents in the Control ND group. Liver discoloration was observed in all the MetS groups, and this finding was absent in the Control ND group. In addition to this, liver-mottled appearance was only observed in the MetS LD group. The spleen seems to be normal and healthy, but a blunt edge appearance of the spleen was observed only in the MetS HD group compared to all the other groups. Abnormalities in the kidney were observed in the MetS groups (fluid collection, lesion, and discoloration) and none in the Control ND group.

**Figure 11:**
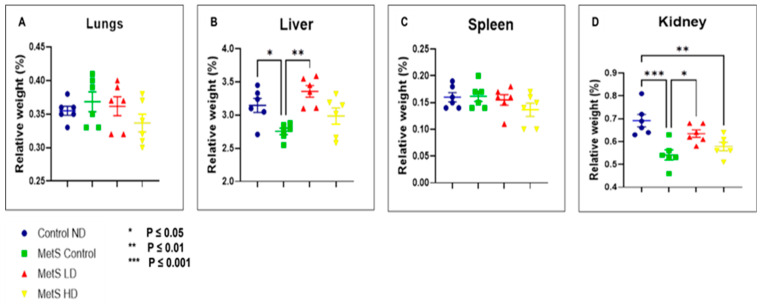
Relative organ weight (%) of (**A**) lungs, (**B**) liver, (**C**) spleen, and (**D**) kidney at week 12. Data were presented as mean ± SEM (*n* = 8 rats per group). A difference at *p* ≤ 0.05 was considered statistically significant. Note: SEM: standard error mean.

Relative organ weight (%) for the lungs, liver, spleen, and kidney were calculated, and the results are shown in Figure 11. No changes were observed in the lungs and spleen in all the groups. In the liver, the MetS Control group showed a statistically significant reduction in relative organ weight (%) between the Control ND group (*p* ≤ 0.05) and the MetS LD group (*p* ≤ 0.01). Meanwhile, for the kidney, the same trend as the liver was observed where a reduction in relative organ weight (%) is statistically significant between the MetS Control group with the Control ND group (*p* ≤ 0.001) and MetS LD group (*p* ≤ 0.05). In addition, a statistically significant (*p* ≤ 0.01) reduction in the kidney relative organ weight (%) was also noted between the Mets HD group and the Control ND group. These results indicated that the low dose of the pooled fetal WJMSCs-derived sEVs preparations has some positive impact on the relative organ weight (%) for both the liver and kidney in the MetS rat.

**Figure 12:**
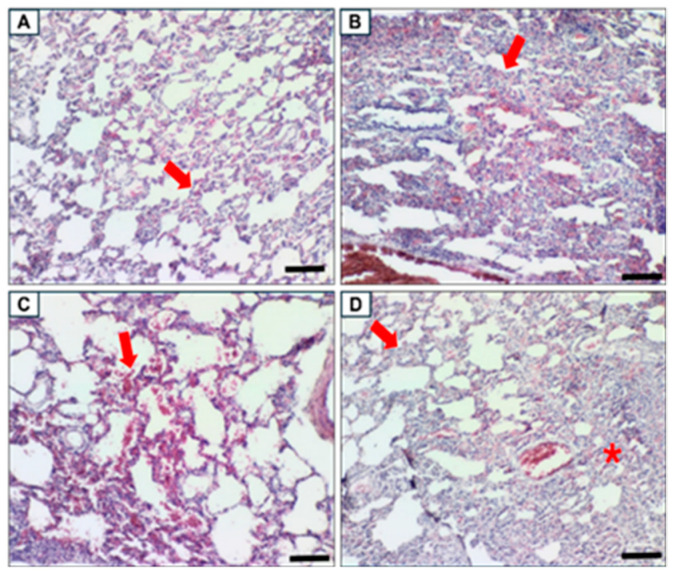
Lung histopathology. The red arrow indicating the histopathology description of each image (**A**) Mild thickening of interalveolar space by mononuclear cells and erythrocytes in Control ND, (**B**) severe thickening of interalveolar space by mononuclear cells and erythrocytes in Mets LD, (**C**) interalveolar hemorrhage in MetS HD, and (**D**) moderate (top left) and severe (* bottom right) thickening of interalveolar space by mononuclear cells and erythrocytes in MetS Control. Scale bar 100 µm, magnification 200×.

**Figure 13:**
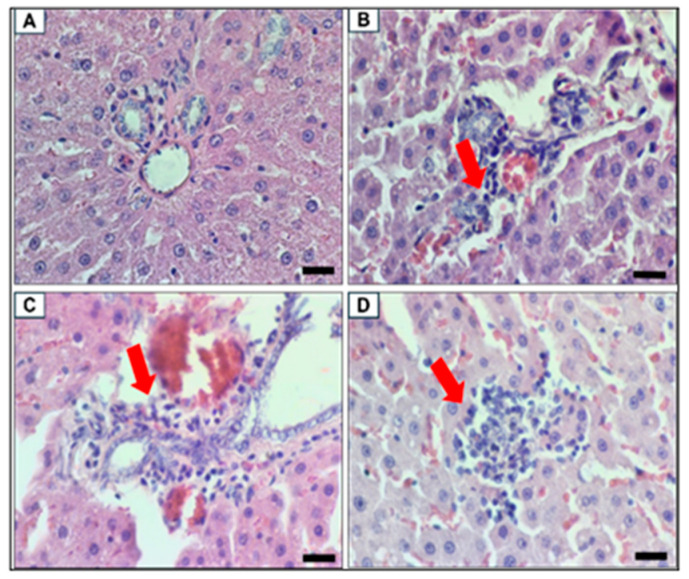
Liver histopathology. The red arrow indicating the histopathology description of each image (**A**) Normal hepatocytes in Control ND, (**B**) portal triad with mild cellular infiltrate in MetS Control, (**C**) portal triad with mild mononuclear cells infiltrate in MetS LD, and (**D**) hepatic parenchyma with focal inflammation in MetS HD. Scale bar 20 µm, magnification 800×.

**Figure 14:**
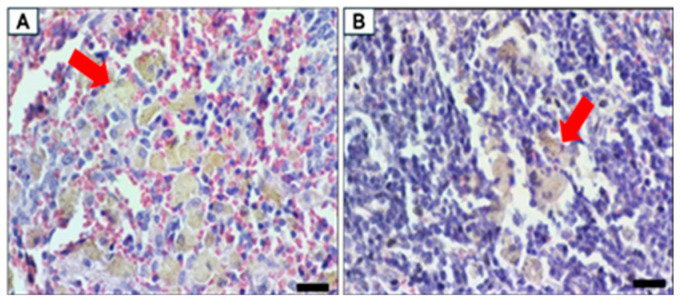
Spleen histopathology. The red arrow indicating the histopathology description of each image Normal spleen. (**A**) Light brown pigment in the red pulp and (**B**) light brown pigment in the white pulp. Scale bar 20 µm, magnification 800×.

**Figure 15:**
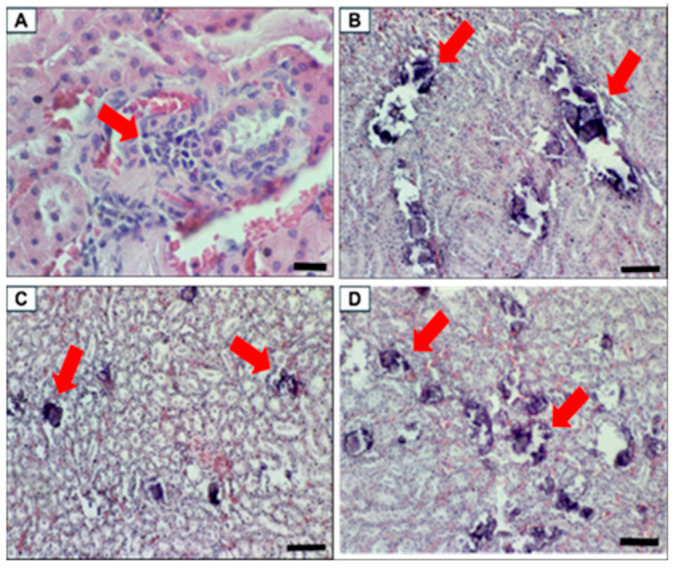
Renal histopathology. The red arrow indicating the histopathology description of each image (**A**) Focal mononuclear infiltrates in the interstitium in Control ND with scale bar 20 µm, magnification 800×, (**B**) severe mineralisation in renal tubules of the outer medulla in MetS Control, (**C**) moderate mineralisation in the renal tubules of outer medulla in MetS LD, and (**D**) moderate mineralisation in renal tubules of the outer medulla in MetS HD. (**B**–**D**) Scale bar 100 µm, magnification 200×.

Histopathological assessment summaries are provided in Table 5, Table 6, Table 7 and Table 8 with the respective images as in Figure 12, Figure 13, Figure 14 and Figure 15. In the lungs, all groups were subjected to moderate-stage interstitial pneumonia, with more severe in the MetS Control group. Mild to moderate hemorrhage condition was also noted in the MetS Control groups compared to only mild hemorrhage in all the other groups. Uniquely, bronchus-associated lymphoid tissue (BALT) hyperplasia was noted in all the MetS groups. Therefore, the pooled fetal WJMSCs-derived sEVs preparations have some protective effects in the lungs for interstitial pneumonia and hemorrhage.

All groups manifested mild cellular infiltrate in the liver, but only the MetS groups showed fatty changes. No inflammation was detected in all the groups. The pooled fetal WJMSCs-derived sEVs preparations had no histopathological changes in the livers of the treated groups compared to the control groups except fatty changes in the MetS groups.

The spleen showed no abnormal histopathological condition, and the presence of light brown pigment in the red and white pulp of the follicles was also noted in all the groups.

All the MetS groups showed mineralisation in the kidney with moderate to severe condition in the MetS Control group and mild to moderate in the EVs treated groups. Interstitium cellular infiltrate was not observed in all the groups, but kidney congestion was discovered in all the groups. Improvement in kidney mineralisation was observed with the pooled fetal WJMSCs-derived sEVs preparations administration on the treated groups in the MetS rat.

## 5. Discussion

Efficacy preclinical study provides the benefits of the potential drug candidates for the intended disease target. A well-designed efficacy study is important for regulatory approval and risk evaluation of the potential drug candidate when tested in clinical trials involving human subjects. Most efficacy studies involve in vivo studies since certain diseases are very complex in nature, and it is best to verify them in the biological system rather than using an in vitro model. Every important aspect of efficacy needs to be analyzed comprehensively to see the overall effects, both intended and unintended [42]. Early determination of any serious adverse effects could save costs in the drug development process too [43].

Using the HFHF diet, we developed a MetS rat model using male SD rats in a 16-week induction period with a success rate of 96% (24 out of 25 rats). Demonstrating the best experimental animal that simulates human ailments is crucial for a successful efficacy study [44,45]. MetS is a type of non-communicable syndrome, and the generally identified causes are sedentary lifestyle and type of diet as discussed in the literature review. Researchers used many approaches to induce and establish MetS in animals, such as diet management, using genetically modified rats, and induction using chemical drugs. Our approach was diet management to induce MetS in animal models, representing the main cause of MetS in humans [46]. Feeding the rats with an HFHF diet to induce MetS tends to have similarities with humans regarding glucose intolerance, elevated blood glucose, dyslipidaemia, and insulin resistance (IR) [45]. HF diet (30–70%) causes an increase in blood glucose, dyslipidemia, elevated free fatty acids, and very low-density lipoprotein in the blood [47,48]. The only disadvantage of using diet management in MetS animal model induction is that it takes longer than using genetically modified rats or chemical drugs. Selection of the type of rat also plays a role, and we used SD rats where their food consumption rate is higher, resulting in weight gain and impaired lipid metabolism [49]. Gender selection of the SD rats also plays a role in MetS study, where male rats are commonly used in MetS studies [50] compared to female rats, where it is best to study specific AT-related research like brown AT [51]. HF diet consumption in animals tends to increase visceral fat and leptin concentration; therefore, this type of diet is suitable for MetS induction, which mimics human MetS condition [52,53]. According to a review by Rodríguez-Correa [28], different severity levels of MetS were produced with different types of diet; HF and HF-high carbohydrate diets produced the severe form of MetS compared to high carbohydrate diet that produced the mild form of MetS. Fructose causes metabolic abnormalities by hepatic lipogenesis through the intermediates for lipid synthesis and fatty acid oxidation inhibition [54,55]. Furthermore, fructose usage was also reported to cause IR and glucose intolerance in rats [56]. This makes 20–30% (*w*/*v*) fructose solution a good choice to combine with the HF diet for the MetS induction in rats [57,58]. The animal’s age also contributes to the establishment of the MetS model since metabolism rates are different at different stages of animal life. It was reported in the research [59] that starting the induction of MetS in rats at the age of 3 weeks and by 4–6 weeks, MetS manifestation was seen drastically compared to induction at 8 weeks, which is the age of an adult rat. To sum up, in our study, we started the HFHF diet at the age of 10 weeks for the male SD rats where we used 35% HF and 30% (*w*/*v*) fructose solution [36,54].

Only the HFHF group displayed morbidity and mortality symptoms with more prominent stress/poor grooming and dyspnea. These symptoms are well related to MetS stress because of the increase in BW and poor grooming due to rough stained coat accumulation of porphyrin [60] around the nose and eyes of the HFHF group rats. Some rats in this group also manifested weight loss, lethargy, and poor response to stimuli, as reported previously [36,60].

In this study, pooled fetal WJMSCs-derived sEVs preparations showed no reduction in AC in the MetS rats. AC is known as waist circumference in humans and is defined as the smallest circumference between the costal margin and iliac crest [61]. Another study in humans defines it as the level midway between the lower rib margin and the iliac crest to the nearest 0.5 cm [62]. The differences in measuring the waist circumference due to different protocols may cause inaccurate results [63] of this simple measurement, which may provide valuable information in diagnosis. The term waist circumference is interchangeably used with AC in animal settings. In animal studies, the AC measurement is not standardized, with few definitions, such as the largest part of the abdominal [64], the circumference of the midpoint between the anterior and posterior legs [65], and the anterior to the forefoot [66]. The obvious differences among researchers in AC measurement may hinder experiment reproducibility and understanding of this MetS parameter. To our knowledge, no study reported the effects of EVs on AC in MetS. Adipose tissue is a metabolically active endocrine organ and has the capability to interfere with biological lipid and glucose metabolism. In a recent study [67], it was found that the removal of epididymal white adipose tissue (EWAT) caused a significant positive impact on insulin resistance, serum insulin, hepatosteatosis, inflammation, and oxidative stress. This is deemed an extraordinary effect of EWAT as one of the metabolically active endocrine organs. Visceral fats are mostly located in the abdominal area and significant changes were not observed with the sEVs treatment in our study and this suggests the involvement of EWAT in MetS as mentioned in the previous studies [67,68].

In this study, it can be concluded that the pooled fetal WJMSCs-derived sEVs preparations have a positive impact on the fasting lipid parameters in MetS rats and is in line with other research [69,70]. However, we found that the impact of sEVs on these parameters is dose-dependent in our study. The high dose causes a reduction in LDL and triglyceride levels with an increase in HDL levels. Meanwhile, the low dose causes a reduction in both CHO and LDL levels. Uniquely, both doses were found to reduce the LDL level, meaning the reduction in LDL level in MetS rats is not dose dependent. Both HDL and triglycerides, as the MetS parameters, were improved with the high dose of pooled fetal WJMSCs-derived sEVs preparations.

Reduction in FBG in MetS rats was observed with the pooled fetal WJMSCs-derived sEVS preparations in my study and in line with another research using 100 µg, 500 µL of exosomes [71], and using 10 mg/kg BW of exosomes [72]. However, in our study, the low dose showed a reduction in FBG compared to the high dose of the pooled fetal WJMSCs-derived sEVS preparations starting from week 6. In OGTT, if the baseline FBG (0 min) is significantly lowered compared to the blood glucose level at 120 min, it shows glucose impairment [73]. In our study, none of the sEVs-treated groups showed better glucose tolerance than the baseline after the treatment started. Therefore, in our study, there is no significant association between the pooled fetal WJMSCs-derived sEVs preparations and the improvement of glucose tolerance in MetS rats. Fasting serum insulin and IR HOMA score results in our study showed that the pooled fetal WJMSCs-derived sEVs preparations have no impact on both these parameters and contradict with other studies [72,74] where EVs reduced the serum insulin level and IR HOMA score after treatment. In week 16, the fasting serum insulin levels were unusually high for all the groups, and we could not find a reasonable explanation for this observation. In our study, we found no significant differences in fasting serum insulin levels post sEVs treatment and subsequently, the same observation with the IR HOMA score since this parameter was derived from the serum fasting insulin levels. The gold standard method used in the clinical diagnosis of IR [75] is the hyperinsulinemic-positive glucose clamp test (HEGC). However, this method is less utilised due to its difficulties in handling and drawbacks. Recent studies [76,77] suggested more reliable biomarkers for IR such as amino acids (branched-chain amino acids and glycine). Fasting blood glucose is one of the key parameters for MetS and this parameter was found to be improved with the sEVs treatment. IR is not a parameter used for clinical diagnosis of MetS and in our study, we found no significant results with fasting serum insulin and the IR HOMA score, suggesting other more reliable biomarkers for the diagnosis of IR such as the branched-chain amino acids (isoleucine, leucine, and valine) and glycine.

According to BP monitoring results, pooled fetal WJMSCs-derived sEVs preparations reduced BP in MetS rats with low dose and high dose acting differently on the SBP and DBP. Overall, there is a significant reduction in BP in MetS rats, and our findings are in line with other studies [78] that use EVs to reduce BP and also in pulmonary hypertension [79]. Based on these results, the action of pooled fetal WJMSCs-derived sEVs preparations on both SBP and DBP was dose dependent.

Several parameters (BW, BL, BMI, food consumption, fluid intake, AC, cholesterol, HDL, triglyceride, serum insulin, and IR HOMA score) have shown significant differences between groups at week −16. Before week −16, two weeks of the acclimatization step were included where the diets for the MetS group were slowly adapted with the high-fat diet and gradually mixed with the standard rat diet so that the rats would be acclimatized. All measurements were started from week −16 and in our opinion, the two-week acclimatization period before the start of the MetS induction period may have caused significant differences between the groups. However, we could not find any literature to support this.

CRP is a widely studied nonspecific inflammation marker. The CRP level is used clinically to diagnose a broad spectrum of inflammation from microbial, tissue injury, and neurodegenerative during the acute phase and recovery stage. Both in acute and chronic inflammation, CRP levels are elevated in biological systems [80]. CRP is also associated with endothelial dysfunction, as reported [81] to forecast cardiovascular-related disorders. Banait concluded that there was no correlation between CRP and MetS, and another research [82,83] also concluded that CRP has a limited capacity to conform to MetS. Meanwhile, another study [84] found a positive link between CRP level and MetS in women and not men. In this study, we found that there are no statistically significant differences in CRP levels after being treated with the pooled fetal WJMSCs-derived sEVs preparations in MetS rats. New biomarkers for inflammation in MetS such as uric acid could be explored in future rather than using CRP which is a general inflammation marker and found to be less useful in MetS [85].

In the results part, we only displayed the leptin and adiponectin results at week 12 (endpoint) because we were experiencing an undetected level of leptin for weeks −16, 0, and 6 using the leptin ELISA kit from Elabscience. Therefore, we used a different brand of leptin ELISA kit from R&D Systems at week 12 to quantify the leptin level. As decided earlier, we were supposed to compare the data for both leptin and adiponectin at each time point. Unfortunately, we do not have the data on leptin levels from weeks −16 to 6 and repeating the leptin levels determination with the new ELISA kit is impossible since we do not have a spare serum for the analysis. Therefore, analyzing the results of the concentration of leptin and adiponectin at different time points was not performed and we only analyzed it at week 12 (endpoint analysis). Leptin levels in the Control ND group were significantly lowered compared to the MetS Control group, and this finding is in line with other studies [82,86,87]. However, our results for the sEVs-treated groups were the opposite, where the levels were significantly higher than those of the MetS Control groups. It showed that the pooled fetal WJMSCs-derived sEVs preparations had no improvement in MetS rats’ leptin levels.

Meanwhile, with adiponectin as anti-inflammatory adipocytokine, significant differences in the level were observed between the sEVs-treated groups only. No significant association between the sEVs-treated group and MetS Control was observed. These results showed that the pooled fetal WJMSCs-derived sEVs preparations have no impact on MetS rats. Most of the studies supported the relationship between leptin and adiponectin with MetS; however, a few studies [83,88,89,90] found that only leptin and the ratio of leptin/adiponectin are utilized to forecast MetS. In addition to leptin and adiponectin, other adipocytokines like omentin and chemerin have been widely studied to investigate their relationship with MetS [91]. A recent study [92] mentioned the presence of two obesity subtypes, metabolically healthy and non-healthy, where the leptin and adiponectin levels were found to be non-significant in both obesity subtypes. Even though adiponectin is an anti-inflammatory substance, it was also reported to have higher levels in autoimmune disorders like type 1 diabetes mellitus, rheumatoid arthritis, systemic lupus erythematosus, and inflammatory bowel disease [93]. Further comprehensive research is warranted to explore and elucidate the complex pathological pathways of these adipocytokines in the future for different types of diseases.

Postmortem evaluation of harvested organs provides pathological input in research dealing with animal study. Necropsy information is crucial in the assessment of the disease state and severity of the disease from general and up to the cellular level. It involves gross necropsy by visual inspection of organs for structural abnormalities and histopathological assessment, where the processed tissue is stained and visualized under a microscope [94,95]. In the lungs, a sign of recovery was observed in the pooled fetal WJMSCs-derived sEVs preparations-treated group for hemorrhages and inflammation compared to the MetS Control group. MSCs-derived EVs have been widely studied and reviewed [96,97,98] and found to have a positive impact on lung-related injury, inflammation, and ailments. BALT hyperplasia was observed only in the MetS groups, and this finding was absent in the Control ND group. BALT develops due to exposure to external factors such as allergens, antigens, microbial agents, or airborne material [99]. The appearance of BALT as an ectopic lymphoid tissue caused by lung inflammation [100] and a hallmark of chronic inflammatory lung diseases such as interstitial lung disease or chronic obstructive pulmonary disease [101]. From our study, we suggest that improvements in BALT were noted in the MetS rat model groups that were treated with sEVs, and there were improvements in lung function related to chronic inflammation. Mild cellular infiltrates were observed in all the liver samples with fatty changes only in all the MetS groups. IR and fatty liver have a compelling relationship [99,102,103], but in our study, serum insulin levels were found to be not statistically significant in all groups. Light brown pigments were observed in both the red and white pulp within spleen follicles in all the groups, and no abnormalities were associated with this histopathology observation. This finding is more related to the sex, genetics, and age of the animal. This light brown pigment is developed from the catabolism activities in parenchyma [104]. In our study, we only used the male SD rats and, therefore, were not affected by these findings [105,106]. Kidney samples in all the MetS groups showed mineralisation with a more severe form in the MetS Control group. Fetal pooled WJMSCs-derived sEVs preparations have protective properties in the MetS LD and MetS HD groups, where moderate to severe mineralisation was only observed in the MetS Control group. Renal mineralisation was not observed in our safety study, and therefore, it can be hypothesized that it occurs only in MetS rats (disease). However, no information was available in the literature regarding the relationship between renal mineralisation and MetS. Kidney mineralisation is associated with the early stages of chronic kidney disease [107] and from our results, we suggested that the sEVs preparations have restored kidney functions. Relative organ weight (%) in the liver and kidney showed a statistically significant increase between the MetS Control group and the MetS LD group; meanwhile, statistically significant reduction was observed between the Control ND group and the MetS Control group. These results showed that the low dose of pooled fetal WJMSCs-derived sEVs preparations has a protective effect on the liver and kidney in MetS rats.

EVs are known to transfer complex information from their cargo to target cells and induce paracrine activities in target cells. In addition, EVs have also been proven to release active ligands from their membrane or dock to the target cells’ membrane to cause biological functional activities. EVs also maintain the normal homeostasis of the normal cells and induce cell response under the pathophysiological condition of the injured or inflamed cells such as adipocytes in the MetS. As reported previously, the target site and cellular response depend on the EVs dose [108]. At physiological levels, the EVs were circulating in relatively lower doses compared to doses of EVs used in both in vitro and in vivo studies. The cellular response due to EVs is influenced by the cell sources, types of tissues, and body fluids. A study reported that high doses of EVs caused the downregulation of the exocytosis process and increased lysosomal activities in target cells by repressing cell membrane trafficking in vivo. Furthermore, high doses of EVs caused an increase in EV-borne transcripts in the target cells [25]. Meanwhile, the low doses of EVs induced specific and greatest transcriptional response and mediated surface protein signalling activities. In another study, the low doses of EVs decreased protein expression compared to the high doses [26]. Again, it depends on the cell targets, disease conditions, and the presence of cellular proteins in the disease state such as cytokines which communicate with the EV cargo contents and also with the EV surface protein. MetS syndrome is caused by chronic low-grade inflammation due to adipocyte dysfunction, which has complex and multifaceted pathophysiological conditions. The low and high doses of EVs cause different mechanisms, for example, direct protein from the cargo intervention, nuclei acid transcription activities, or the interference of the surface protein marker of the EVs with the target cells which induces the immunomodulatory and anti-inflammatory activities on the target cells. The high dose seems to influence lipid metabolism activities, and the low dose reduces the fasting blood glucose through the normalisation of insulin resistance activities in the muscle cells. Meanwhile, both doses may play a role in the repair and regeneration of endothelial cell dysfunctions which causes the improvement in blood pressure. In our opinion, if there is no dose-dependent cellular response, probably all activities related to the normalisation of homeostasis and repairing at almost the same rate such as transcriptional and lysosomal activities.

## 6. Conclusions and Limitation

In conclusion, in this efficacy study, we evaluated the efficacy of the pooled human fetal WJMSCs-derived sEVs preparations IV injection on MetS rats and the relationship of the leptin–adiponectin axis in MetS. The effects of two different doses were evaluated, the low dose (3 × 10^9^ particles/rat) and high dose (9 × 10^9^ particles/rat) injected intravenously with the frequency of every three weeks. The evaluated parameters in this efficacy study are physical measurement (BW, BL, AC, BMI, food consumption, and fluid intake), SBP and DBP, blood glucose (FBG and OGTT), fasting serum insulin with IR HOMA score, fasting serum CRP, fasting lipid profile (CHO triglyceride, HDL and LDL), necropsy for lungs, liver, spleen, and kidney (gross necropsy, relative organ weight, and histopathology assessment). Fasting serum leptin and adiponectin were evaluated for the relationship between the leptin–adiponectin axis with MetS. Among all these parameters in this efficacy study, only five were the MetS parameters: AC, BP, FBG, triglycerides, and HDL. The pooled human fetal WJMSCs-derived sEVs preparations have no significant reduction in AC in MetS rats after treatment. For BP, the pooled human fetal WJMSCs-derived sEVs preparations significantly reduced the BP in MetS rats. In addition, the low dose of the pooled human fetal WJMSCs-derived sEVs preparations reduced the FBG in MetS rats. A high dose of these sEVs preparations also reduced the triglycerides level after treatment in MetS rats. Meanwhile, for the HDL level, the high dose of the pooled human fetal WJMSCs-derived sEVs preparations increased the HDL level after treatment in MetS rats. Regarding the overall outcome of this efficacy study, we found that the pooled human fetal WJMSCs-derived sEVs preparations administered intravenously every three weeks only improved four out of five MetS parameters in the MetS rat model. Some of the parameter’s responses were dose dependent. In this study, the relationship between the leptin–adiponectin axis and MetS could not be established due to very limited data, and the available data were only for the endpoint comparison at the end of the study at week 12. Based on these limited data, preliminarily it can be concluded that the pooled fetal WJMSCs-derived sEVs preparations have no impact on either the leptin or adiponectin in MetS rats. No apparent adverse effects were detected after pooled human fetal WJMSCs-derived sEVs preparations were administered in the MetS rats. In addition, pooled human fetal WJMSCs-derived sEVs preparations showed inflammation and hemorrhage recovery in the lungs and reduced the severity of mineralisation in the kidney of MetS rats, probably through their immunomodulatory and anti-inflammatory properties. To sum up, this study provides insight into the management of MetS using the pooled human fetal WJMSCs-derived sEVs preparations via the IV route of administration every three weeks. This study also provides an efficacy profile and basic groundwork for the future application of EVs as nanomedicine in clinical studies.

Several limitations have been identified in this study. The induction of MetS in rats was started at the age of ten weeks for the rats, and it took 16 weeks to develop MetS in rats using the HFHF diet. Therefore, the rats were ageing when the treatment was started. As per the literature, induction of MetS using the diet method could be started as early as three weeks of rat age. This study also could include female rats to evaluate the effects of gender on the treatment of MetS using human fetal WJMSCs-derived sEVs preparations. We could not repeat the leptin determination from weeks −16 to 6 due to the problem associated with the undetected level of leptin using the ELISA kit from Elabscience. However, we did not have spare serum to repeat these tests using the different brands of leptin ELISA kits. Since some of the parameter responses were dose dependent, a more detailed experiment could be performed to evaluate different doses, and this may provide some insight into the mechanism of action and specific dosing strategy in clinical application.

## Figures and Tables

**Table 1:** Summary of rats monitoring, frequency, and parameters/organs.

Type	Frequency	Parameters/Organs
Physical measurement	Every 6 weeks (−16, 0, 6, 12)	BW, BL, AC, BMI, food consumption, and fluid intake
BP	Every 6 weeks (−16, 0, 6, 12)	SBP and DBP
Blood glucose	Every 6 weeks (−16, 0, 6, 12)	FBG and OGTT
Serum insulin	Every 6 weeks (−16, 0, 6, 12)	Fasting serum insulin
Serum CRP	Every 6 weeks (−16, 0, 6, 12)	Fasting serum CRP
Serum leptin and adiponectin	In week 12 only	Fasting serum leptin and adiponectin
Lipid profile	Every 6 weeks (−16, 0, 6, 12)	Fasting serum CHO, LDL, HDL, and triglycerides
Insulin resistance (IR) and glucose tolerance	Every 6 weeks (−16, 0, 6, 12)	IR HOMA score
Necropsy	End of week 12	Gross pathological evaluation of lungs, liver, spleen, and kidney with relative weight of the organs
Histopathological	End of week 12	Assessment of lungs, liver, spleen, and kidney

Note: BW: body weight; BL: body length; AC: abdominal circumference, BMI: body mass index; BP: blood pressure; SBP: systolic blood pressure; DBP: diastolic blood pressure; FBG: fasting blood glucose; OGTT: oral glucose tolerance test; CRP: C-reactive protein; CHO: cholesterol; LDL: low-density lipoprotein; HDL: high-density lipoprotein; IR HOMA: Insulin Resistance Homeostatic Model Assessment.

**Table 2:** MetS parameters’ normal average, standard deviation, and range derived from the Control ND group.

MetS Parameters	Average	Standard Deviation (SD)	Range
Systolic BP (mmHg)	107.6	13.7	93.9–121.3
Diastolic BP (mmHg)	74.8	10.2	64.6–85.0
FBG (mmol/L)	5.5	0.53	4.97–6.03
Triglycerides (mmol/L)	0.39	0.14	0.25–0.53
HDL (mmol/L)	0.65	0.05	0.6–0.7
AC (cm)	21.6	0.58	21.02–22.18

**Table 3:** MetS scoring summaries for all the HFHF rats.

MetS Score	Number of HFHF Rats
1/5	0
2/5	1
3/5	3
4/5	16
5/5	5
Total	25

**Table 4:** Gross necropsy evaluations of the harvested organ.

Group	Observations			
	Lungs	Liver	Spleen	Kidney
Control ND	Mild congestion (*n* = 4)	Normal	Normal	Normal
MetS Control	Normal	Discoloration (*n* = 1)	Normal	Discoloration (*n* = 1)
MetS LD	Mild congestion (*n* = 1)	Discoloration and mottled appearance (*n* = 3)	Normal	Lesion (*n* = 1) Fluid collection (*n* = 1)
MetS HD	Normal	Discoloration (*n* = 2)	Blunt edge appearance (*n* = 1)	Lesion (*n* = 1)

**Table 5:** Summary of histopathology assessment of the lungs.

Assessment Parameter	Control ND	MetS Control	MetS LD	MetS HD
Inflammation (interstitial pneumonia)	Moderate	Moderate	Moderate	Moderate
Interstitial pneumonia description	Mild to moderate multifocal thickening	Moderate to severe focal thickening	Moderate multifocal thickening	Moderate multifocal thickening
Hemorrhage	Mild	Mild to moderate	Mild	Mild
Congestion	Yes	Yes	Yes	Yes
Bronchus-associated lymphoid tissue (BALT) hyperplasia	No	Yes	Yes	Yes

**Table 6:** Summary of histopathology assessment of the liver.

Assessment Parameter	Control ND	MetS Control	MetS LD	MetS HD
Inflammation	Absent	Absent	Absent	Absent
Cellular infiltrate	Mild	Mild	Mild	Mild
Other	None	Fatty changes	Fatty changes	Fatty changes

**Table 7:** Summary of histopathology assessment of the spleen.

Assessment Parameter	Control ND	MetS Control	MetS LD	MetS HD
Pigment distribution	Red and white pulp contains light brown pigment	Red and white pulp contains light brown pigment	Red and white pulp contains light brown pigment	Red and white pulp contains light brown pigment

**Table 8:** Summary of histopathology assessment of the kidney.

Assessment Parameter	Control ND	MetS Control	MetS LD	MetS HD
Interstitium cellular infiltrate	Absent	Absent	Absent	Absent
Mineralisation	None	Moderate to severe	Mild to moderate	Mild to moderate
Others	Congestion	Congestion	Congestion	Congestion

## Data Availability

Data are contained within the article.

## Supplementary Materials

The following supporting information can be downloaded at: https://www.mdpi.com/article/10.3390/biom15010044/s1, Table S1: Morbidity and mortality observations of rats throughout the study and Figure S1: Data for week −16.
