# Supplementary material for: Efficacy of Fetal Wharton’s Jelly Mesenchymal Stem Cells-Derived Small Extracellular Vesicles in Metabolic Syndrome
Source: Biomolecules. 2025 Jan 1;15(1):44. doi: 10.3390/biom15010044 (PMC11763124; doi:10.3390/biom15010044)
Supplement: Supplementary file 1 [file biomolecules-15-00044-s001.zip › biomolecules-3313029-supplementary.pdf]

Supplementary Table S1: Morbidity and mortality observations of rats throughout the study.

Observations parameters	Study period (week)		
	0	6	12
Anorexia, weight loss, and/or dehydration	HFHF (*n*=2/group)	HFHF (*n*=2/group)	HFHF (*n*=2/group)
Dyspnea	HFHF only (*n*=7/group)	HFHF only (*n*=8/group)	HFHF only (*n*=8/group)
Prolonged hypothermia or hyperthermia	NIL	NIL	NIL
Stress and/or poor grooming	HFHF only (*n*=8/group)	HFHF only (*n*=8/group)	HFHF only (*n*=8/group)
Lethargy, hunched posture, and inability to rise or ambulate	HFHF only (*n*=3/group)	HFHF only (*n*=3/group)	HFHF only (*n*=3/group)
Poor reflex or irresponsiveness to external stimuli	HFHF only (*n*=3/group)	HFHF only (*n*=3/group)	HFHF only (*n*=3/group)
Tumor growth	NIL	NIL	NIL

NIL: absence of signs and symptoms

Supplementary Figures S1: Data for week -16

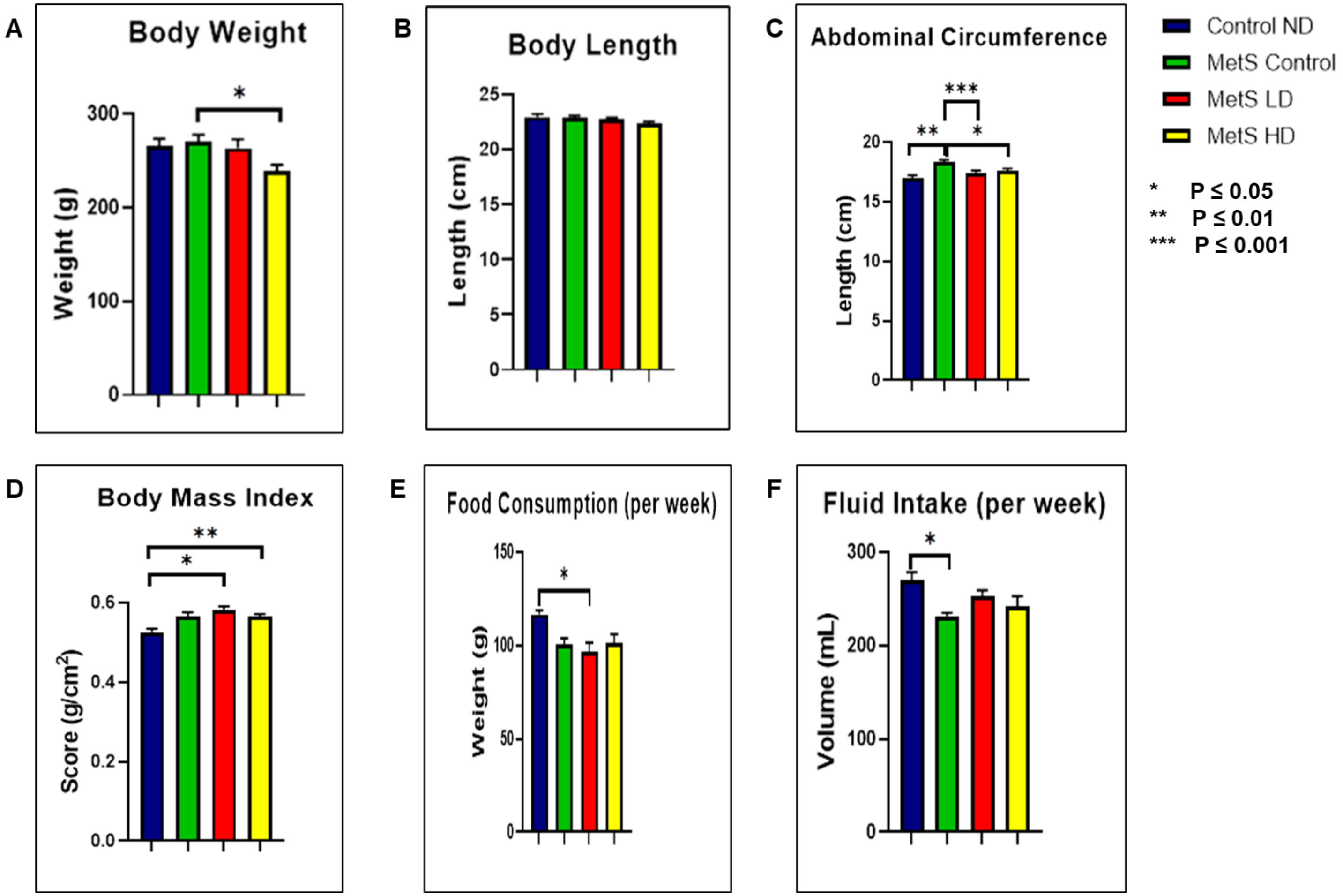

Figure S1.1: Physical measurement at -16 weeks. **[A]** Body weight, **[B]** body length, **[C]** abdominal circumference,**[D]** body mass index, **[E]** food consumption (per week), and **[F]** fluid intake (per week). Data was presented as mean ± SEM (*n* = 8 rats per group). A difference at p ≤ 0.05 was considered statistically significant

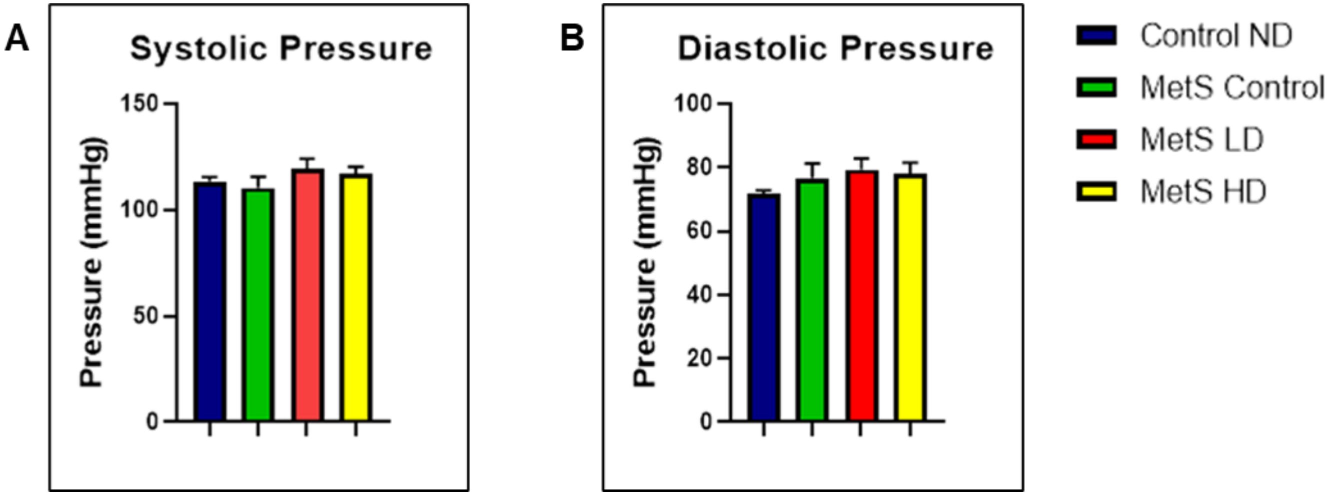

Figure S1.2: Blood pressure at -16 weeks. **[A]** Systolic pressure and **[B]** diastolic pressure. Data was presented as mean ± SEM (*n* = 8 rats per group). A difference at $p \leq 0.05$ was considered statistically significant.

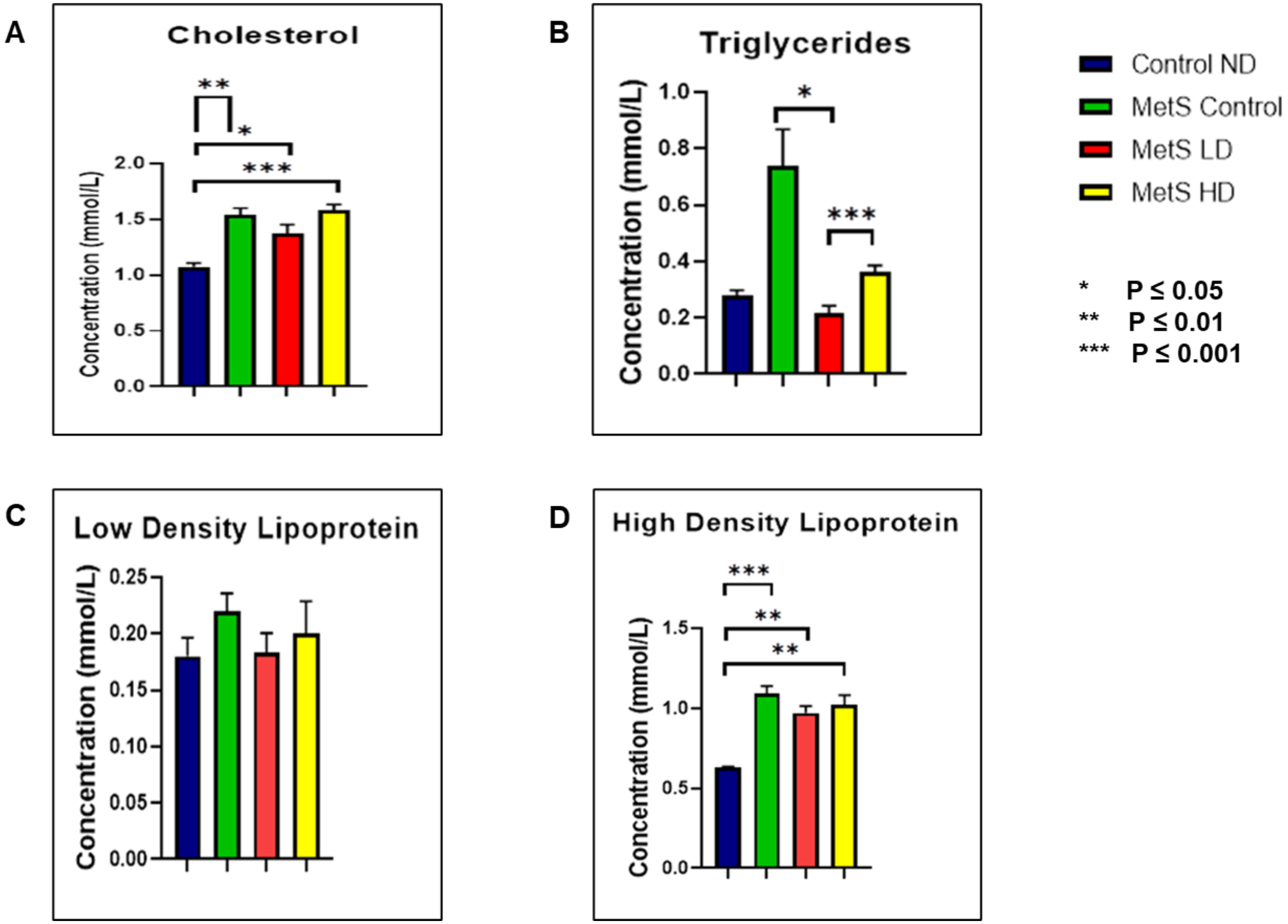

Figure S1.3: Serum fasting lipid profile at -16 weeks. **[A]** Cholesterol, **[B]** triglycerides, **[C]** low-density lipoprotein,**[D]** high-density lipoprotein. Data was presented as mean ± SEM (*n* = 8 rats per group). A difference at $p \leq 0.05$ was considered statistically significant.

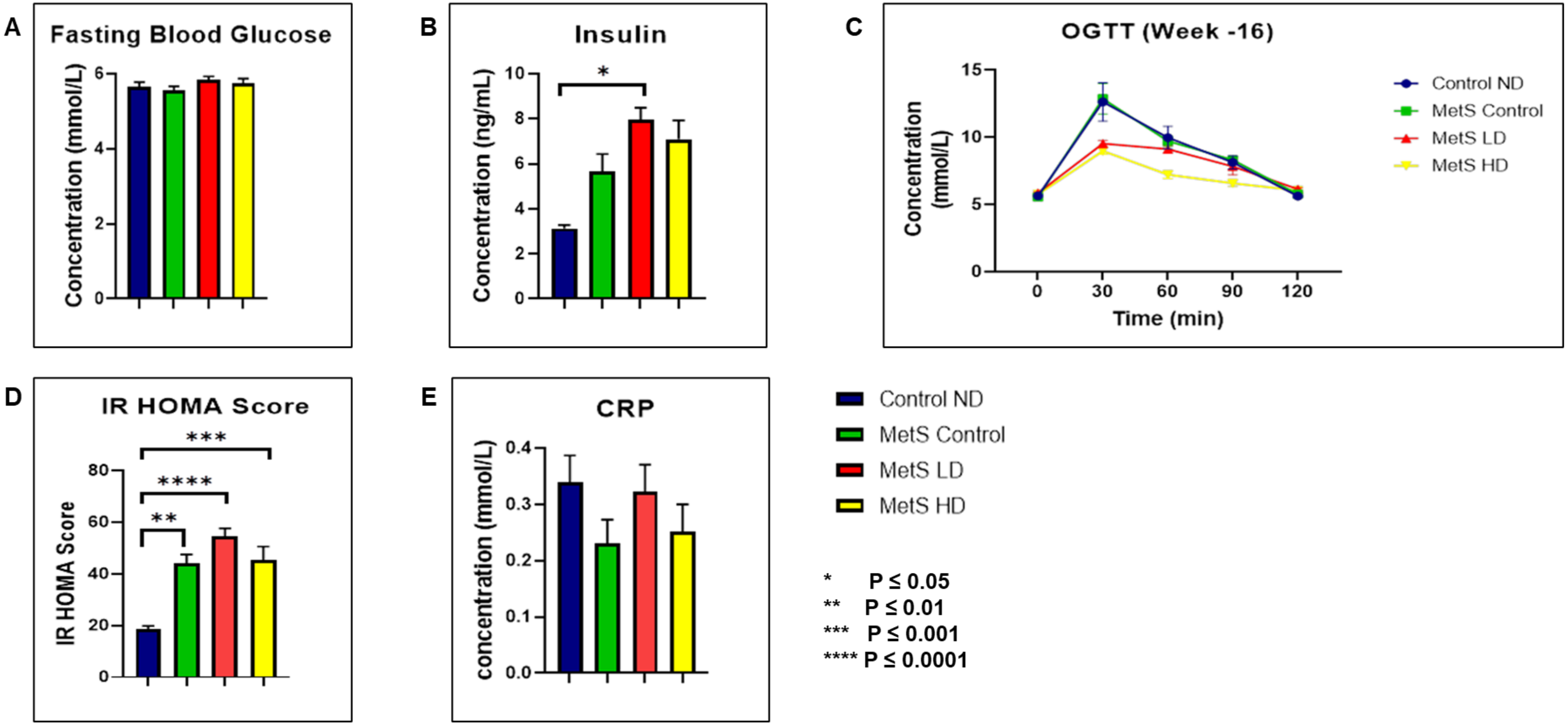

Figure S1.4: Parameters at -16 weeks. **[A]** Fasting blood glucose, **[B]** fasting serum insulin, **[C]** oral glucose tolerance test,**[D]** IR HOMA score, and **[E]** fasting serum CRP. Data was presented as mean ± SEM (*n* = 8 rats per group). A difference at p ≤ 0.05 was considered statistically significant
